# Storage of boar semen at 16-18 °C in the long-term commercial extender prepared with deionized water or nanowater

**DOI:** 10.21451/1984-3143-AR2019-0023

**Published:** 2019-11-18

**Authors:** Joanna Szymanowicz, Tomasz Schwarz, Maciej Murawski, Martyna Małopolska, Zdzisław Oszczęda, Ryszard Tuz, Jacek Nowicki, Pawel Mieczyslaw Bartlewski

**Affiliations:** 1 Agricultural University of Cracow, Department of Animal Biotechnology, Kraków, Poland; 2 Agricultural University of Cracow, Department of Swine and Small Animal Breeding, Kraków, Poland; 3 National Institute of Animal Production, Department of Pig Breeding, Balice, Poland; 4 Nantes Nanotechnology Systems, Bolesławiec, Poland; 5 University of Guelph, Ontario Veterinary College, Department of Biomedical Sciences, Guelph, Canada

**Keywords:** pig, sperm, liquid storage, semen extender, nanowater

## Abstract

The aim of this study was to assess the usefulness of nanowater (NW; water declusterized using cold plasma treatment) as a diluent for a commercial boar semen extender during the 15-day storage (Days 1 to 15) at 16-18 °C. Ejaculates collected from 8 boars were subjected to the standard evaluation and then diluted in the extender prepared with deionized water (DW) or NW to a final concentration of 3×10^9^ spermatozoa/ml. The proportion of defective spermatozoa increased (P<0.05) from Day 10 to Day 15 of storage (22.8±16.6% to 41.8±26.4% in DW group and 18.6±11.7% to 34.8±25.4% in NW group) and it was significantly greater in DW group compared with NW group on Days 5 and 10 due mainly to a greater (P<0.05) number of mid-piece defects in semen stored in the DW-containing extender. Sperm progressive motility decreased (P<0.05) in both groups between Days 2 and 6, Days 6 and 10, and Days 10 and 12, whereas the percentage of motile spermatozoa declined (P<0.05) to Day 14 only in NW group. Sperm motility was greater (P<0.05) in NW group compared with DW group from Day 5 to Day 13. A decline in sperm progressive motility below 40% in all semen samples occurred by Day 11 in DW group and by Day 12 in NW group. The mean survival time of sperm at 37 °C *ex situ* was greater in NW group than in DW group on Day 5 (314±87 min compared with 284±87 min) and Day 10 (223±34 min compared with 182±27 min; NW group compared with DW group, respectively). There were no differences (P>0.05) between the two groups in the concentrations of alkaline phosphatase and aspartate aminotransferase in semen extender. To summarize, the use of NW as an extender diluent exerts cytoprotective effects on boar spermatozoa and delays a decline in sperm progressive motility.

## Introduction

First successful artificial insemination (AI) in pigs was performed by a Russian biologist Elie Ivanov early in the 20^th^ century (Ivanow, 1907, 1922; Knox, 2016). Milovanov (1938) developed the first extenders (glucose/sulphate- and glucose/tartrate-based) for boar semen shipment and use in the field. In the 2000s, more than 99% of the estimated 19 million AIs performed each year in commercial swine operations all over the world utilize semen that has been extended in the liquid state, and then transported and/or stored at 15-20 °C for up to 5 days (Gadea, 2003). The prolonged storage time has adverse effects on porcine sperm viability; therefore, approximately 85% of inseminations are performed within 48 h of semen collection (Gadea, 2003). Farrowing rates between 80 and 85% can be achieved using extended boar semen up to 48 h post-ejaculation but longer storage may be associated with a significant reduction in piglet productivity of inseminated sows (Boe-Hansen et al., 2008). Although several attempts have been made to extend the duration of liquid semen preservation beyond 3 days (long-term storage) without any negative effects on fertility, the results to date have been inconsistent and unsatisfactory (Gadea, 2003).

Changes in the structure and function of boar spermatozoa during liquid storage of semen resemble those that occur during natural ageing processes (Johnson et al., 2000). Sperm deterioration and mortality are due mainly to initial cold-shock, dilution of seminal plasma constituents and/or bacterial contamination. Porcine spermatozoa are particularly sensitive to cooling because of the low cholesterol: phospholipid ratio of their cell membranes (Bresciani et al., 2017). Semen susceptibility to cold shock results in an irreversible disruption of the cytoplasmic membrane permeability (Althouse et al., 1998) due mainly to alterations in the lipid bilayer (Watson, 2000). Cytoskeletal elements are also very sensitive to temperature changes and cooling may cause a premature depolimerization of cellular actin filaments (Hall et al., 1993). Continued metabolic processes gradually lead to the depletion of nutrients and natural antioxidants, and the accumulation of metabolic end products. The resultant changes in semen parameters, such as pH and osmolality, ultimately alter sperm DNA integrity (Fraser and Strzeżek, 2004). Dilution of boar ejaculates reduces the availability of seminal plasma proteins and other constituents that are essential for sustaining cell membrane conformation (Boe-Hansen et al., 2008). Lastly, various microbial contaminations of the extended semen are detrimental for the survival time of spermatozoa and their overall viability due mainly to the rapid and undesirable changes in pH values of semen storage media associated with bacterial growth (Hofmo, 1991).

Deleterious effects that occur during liquid semen storage can potentially be reduced by the addition of a suitable semen extender (Vyt et al., 2004; Boe-Hansen et al., 2005). Up to date, nanowater (NW; water subjected to cold plasma treatment; Mystkowska and Dąbrowski, 2013) has not been widely used in semen preservation even though its unique properties could be very useful (Murawski et al., 2015). Under normal conditions, water contains aggregates consisting of up to 1,000 molecules (a.k.a. clusters). Exposure to cold plasma causes partial declusterization of water molecular structure and formation of nanoclusters (Mystkowska and Dąbrowski, 2013). This process in turn results in the acquisition of unique physicochemical properties. Water treated in the low-temperature plasma reactor freezes at −67 °C, boils at 187 °C, and has a zero coefficient of thermal expansion at freezing (Niemirowicz and Car, 2012; Mystkowska and Dąbrowski, 2013; Murawski et al., 2015). NW is characterized by low viscosity, high diffusivity, and very low density. Due mainly to a low dielectric constant, NW significantly increases solubility of both organic and inorganic compounds (NW dissolves up to 40% more substances than equal volume of non-declusterized water and it increases the solubility of gases and salts by ~50%, which permits obtaining highly concentrated solutions). Therefore, the use of NW may provide a novel method to increase the availability of various extender constituents to the boar sperm throughout the entire period of liquid storage. NW also possesses antimicrobial properties (Bai et al., 2011; Julák et al., 2012). 

We hypothesized that the addition of NW to a commercially available extender would delay a decline in sperm progressive motility and survivability. The specific aim of this preliminary study was to assess the effects of NW as a semen extender diluent on main morphological, functional and biochemical characteristics of boar semen during the 15 days of storage at 16-18 °C.

## Methods

The present experiment utilized eight sexually mature Polish Large White x Polish Landrace boars housed in the Boar Utilization Facility in Klecza Dolna, Poland (49°53’09.3”N 19°32’02.1”E) and ejaculated once in the month of November. The ejaculates were collected into pre-warmed, hand-held plastic tubes (~30 °C) and subjected to standard evaluation procedures: measurements of volume, sperm concentration and progressive motility. Semen volume was measured in the graded collection flask, sperm concentration was determined in the Bürker chamber, and sperm progressive motility was estimated under a microscope with a warm plate at 37 °C and image magnification of ×200. Additionally, two smears of each ejaculate were stained with the SpermBlue® stain (Microptic S.L., Barcelona, Spain) for subsequent microscopic examinations.

Semen extenders used in this study were prepared by dissolving 40 g of the commercially available boar semen extender (Cronos®; Medi Chimica, Reggio Emilia, Italy) in 1 l of deionized water (DW; Aqua Purificata®; Prolab, Gliwice, Poland) or 1 l of nanowater (NW; Nantes Nanotechnology Systems, Bolesławiec, Poland). An ejaculate from each boar was divided into two equal volumes and diluted with the two extenders at 30 °C until the attainment of a final concentration of 3×10^9^ spermatozoa/ml. Extended semen was loaded into 80-ml vials (insemination doses) and after 4 h of stabilizing at room temperature (in darkness) stored at the controlled temperature of 16-18 °C for 15 days. Each vial was turned over every 8 h (or 3 times a day) throughout the entire storage period to prevent semen sedimentation.

Semen morphology (300 spermatozoa/slide) was evaluated on the 2^nd^, 5^th^, 10^th^ and 15^th^ day of storage using the computer analysis system SCA (Sperm Class Analyzer®; Microoptic S.L., Barcelona, Spain). Sperm motility (percentage of progressively motile sperm) was determined every day until the end of experiment. Thermoresistance sperm test in a water bath (39 °C) was carried out on Days 2, 5 and 10. Semen samples (2 ml) in Eppendorf tubes were placed in a water bath and sperm motility was recorded every 30 min until complete cessation. The cut-off point for comparing sperm motility between control and treatment groups was the attainment of ≤40% of motile spermatozoa, which is the lowest acceptable value permitting the use of a semen dose for commercial AI. Alkaline phosphatase (ALP) and aspartate aminotransferase (AspAT) tests were performed on extender samples every other day until the 6^th^ day of storage, and then every day until Day 15. Supernatants for these analyses were obtained after centrifuging 1.5 ml of semen at 10,000×*g* for 5 min and then kept frozen at −28 °C until analyses at the later date. ALP and AspAT concentrations were measured by a method based on the recommendations of the International Federation of Clinical Chemistry. Enzyme quantities were determined by the kinetic UV method using direct potentiometry, which utilizes ion-selective electrode modules for electrolyte analysis without sample dilution (Murawski et al., 2015). The analytical method used was based on the “dry slide technology” employing the thin film diagnostic laminae (ALP: Abbott Laboratories Poland Ltd., Warsaw, Poland; and AspAT: DiaLab Laboratoria Medyczne, Wrocław, Poland). A biochemical analyzer Vitros® (Ortho Clinical Diagnostics; High Wycomb, UK) was used and all tests were performed at 37 °C. Specific methodological approaches and chemical reactions for ALP and AspAT detection have been described elsewhere (Murawski et al., 2015).

Data were analyzed by two-way repeated measures analysis of variance (RM-ANOVA) using SigmaPlot® (version 11.0; Systat Software Inc., Richmond, CA, USA). All data sets were initially subjected to the normality (Shapiro-Wilk) and equal variance tests. If the raw data failed one or both of those tests, they were converted by log_n_ before analysis of variance. The Holm-Sidak test was used for comparisons of individual means if any of the main effects and/or their interaction was significant; an overall significance was set at P≤0.05. Correlation analyses (Pearson Product Moment Correlation) were performed among the proportions of ejaculate in inseminate doses (extended semen) and all semen characteristics determined in this experiment, in order to examine the potential influence of semen dilution on morphological and functional changes in boar spermatozoa during the 15 days of storage. All results are given as mean±standard deviation (mean±SD) unless otherwise stated.

## Results

Semen characteristics determined immediately after ejaculation are summarized in Table 1. The percentage of abnormal spermatozoa was significantly greater on Day 15 compared with Days 2, 5 and 10 in both DW and NW groups (Table 2). The percentage of sperm with tail defects and proximal droplets increased (P<0.05) from Day 2 to Day 15 in both groups. The percentage of boar spermatozoa with head defects increased (P<0.05) from Day 2 to Day 15 in DW group. The proportion of detached sperm heads was less (P<0.05) on Days 2, 5 and 10 compared with Day 15 in DW group and it was less (P<0.05) on Day 5 than on Day 15 in NW group. There was a 2-5-fold increase (P<0.05) in the percentage of sperm with defective mid-pieces between Days 2 and 15 in DW group and the proportion of spermatozoa with detected mid-piece defects was approximately 6-fold greater (P<0.05) after 15 days of storage compared with Days 2, 5 and 10 in NW group. The percentage of sperm with distal droplets was greater (P<0.05) on Day 15 compared with Days 2 and 5 in boar semen stored in the NW extender. Lastly, the DW group semen exceeded (P<0.05) NW inseminates in the total percentage of defective sperm and the proportion of spermatozoa with mid-piece defects on Days 5 and 10.

**Table 1:** Ejaculate and extended semen characteristics (mean±SD) in eight Polish Large White x Polish Landrace boars.

**Variable**	**Mean values and ranges**
Volume (ml)	218.8±69.8 (160-320)
Semen concentration (×10^6^/ml)	482.1±171.5 (304-847)
Progressive motility (%)	77.5±3.8 (70-80)
No. of insemination doses/ejaculate	26.2±10.5 (12-47)
Ejaculate volume/inseminate volume×100 (%)	9.0±3.1 (4-14)
Defective spermatozoa (total) (%)	15.0±8.0 (5-32)
Head defects (%)	1.9±1.3 (1-4)
Loose heads (%)	0.06±0.07 (0-0.5)
Double head (%)	0.6±0.8 (0-2)
Mid-piece defects (%)	1.9±1.7 (0-4)
Tail defects (%)	7.5±7.8 (2-24)
Double tail (%)	0.06±0.07 (0-0.5)
Proximal droplets (%)	1.8±2.6 (0-6)
Distal droplets (%)	0.9±0.8 (0-2.5)

Ranges of values for each variable are given in parentheses.

**Table 2:** Morphological defect rates (mean±SD) of boar spermatozoa on the 2^nd^, 5^th^, 10^th^ and 15^th^ day of liquid semen storage at 16-18 °C in a commercial, long-term semen extender prepared with deionized water (DW) or nanowater (NW).

**Variable (%)**	**Group**	**Day 2**	**Day 5**	**Day 10**	**Day 15**
Defective spermatozoa (total)	DW	11.2±8.2a	18.8±10.6a*	22.8±16.6a*	41.8±26.4b
	NW	6.1±2.7a	11.3±4.2a*	18.6±11.7a*	34.8±25.4b
Head defects	DW	2.5±1.2a	3.2±1.1ab	4.0±1.8ab	5.9±3.8b
	NW	2.2±1.4	2.5±1.4	4.0±2.1	3.8±2.5
Loose heads	DW	0.7±0.7a	1.0±1.0a	1.0±1.0a	2.2±2.1b
	NW	1.2±1.1ab	0.4±0.5a	1.1±1.3ab	2.4±2.8b
Double head	DW	-	0.6±0.7	-	-
	NW	-	0.4±0.7	-	0.1±0.3
Mid-piece defects	DW	5.4±7.9a	7.1±6.3ab*	7.9±6.6ab*	13.3±12.9b
	NW	1.7±0.9a	1.7±1.0a*	1.9±1.0a*	11.9±12.2b
Tail defects	DW	0.1±0.3a	3.7±4.5ab	5.7±9.6ab	12.4±15.6b
	NW	0.4±0.7a	3.9±4.0ab	7.0±9.7ab	11.1±13.0b
Double tail	DW	0.06±0.17	0.12±0.23	0.25±0.38	0.36±0.70
	NW	0.06±0.17	0.06±0.17	0.06±0.17	-
Proximal droplets	DW	0.9±0.6a	1.8±1.4ab	1.9±1.8ab	3.8±3.0b
	NW	0.6±0.7a	1.2±1.3ab	2.1±2.7ab	3.0±3.8b
Distal droplets	DW	1.9±0.5	1.4±1.0	2.1±2.1	2.8±3.2
	NW	0.5±0.6a	1.9±0.8a	2.4±2.0ab	3.6±2.8b

Within rows, mean values denoted by different letters vary significantly and asterisks indicate significant differences between the two groups within the same day for each variable.

The mean survival time of boar spermatozoa *ex situ* decreased (P<0.05) between Days 2 and 5, and between Days 5 and 10 (Table 3). The time to the first decline in sperm progressive motility to ≤40% decreased (P<0.05) while the duration of the ensuing period until complete demise of all spermatozoa increased (P<0.05) between Days 2 and 5. Significant differences between the two groups in the mean survival time of spermatozoa were recorded on Days 5 and 10 (Table 3); on both days, the survival time was greater by ~0.5 h in NW group. A decline in sperm progressive motility to ≤40% occurred, on the average, 70 and 35 min earlier in DW compared with NW group on Days 5 and 10, respectively (P<0.05). On Day 15 of storage, sperm motility was ≤5% in all semen samples tested.

**Table 3:** Major characteristics of boar semen viability (mean±SD) determined *ex situ* on Days 2, 5 and 10 of the liquid storage in a commercial semen extender prepared with deionized water (DW) or nanowater (NW).

**Variable**	**Group**	**Day 2**	**Day 5**	**Day 10**
Survival time (min)	DW	372.5±18.7a	283.7±86.6b*	181.9±27.1c*
	NW	383.1±27.9a	313.7±87.2b*	223.1±34.4c*
Time reaching ≤40% motility (min)	DW	206.2±58.8a*	63.7±76.0b*	[90, 90]
	NW	274.5±59.5a*	99.4±73.0b*	[90, 60, 60, 90]
Time from reaching ≤40% motility to demise (min)	DW	166.2±49.1a	220.0±41.7b	[75, 90]
	NW	135.6±46.7a	214.4±48.3b	[150, 75, 135, 180]

Within rows, mean values denoted by different letters are significantly different and asterisks indicate significant differences between the two groups within the same day for each variable. On Day 10, semen progressive motility at the outset of the survival trial test was ≥40% only in two inseminates in DW and four inseminates in NW group (values given in brackets); these data were excluded from further statistical analyses.

**Figure 1:**
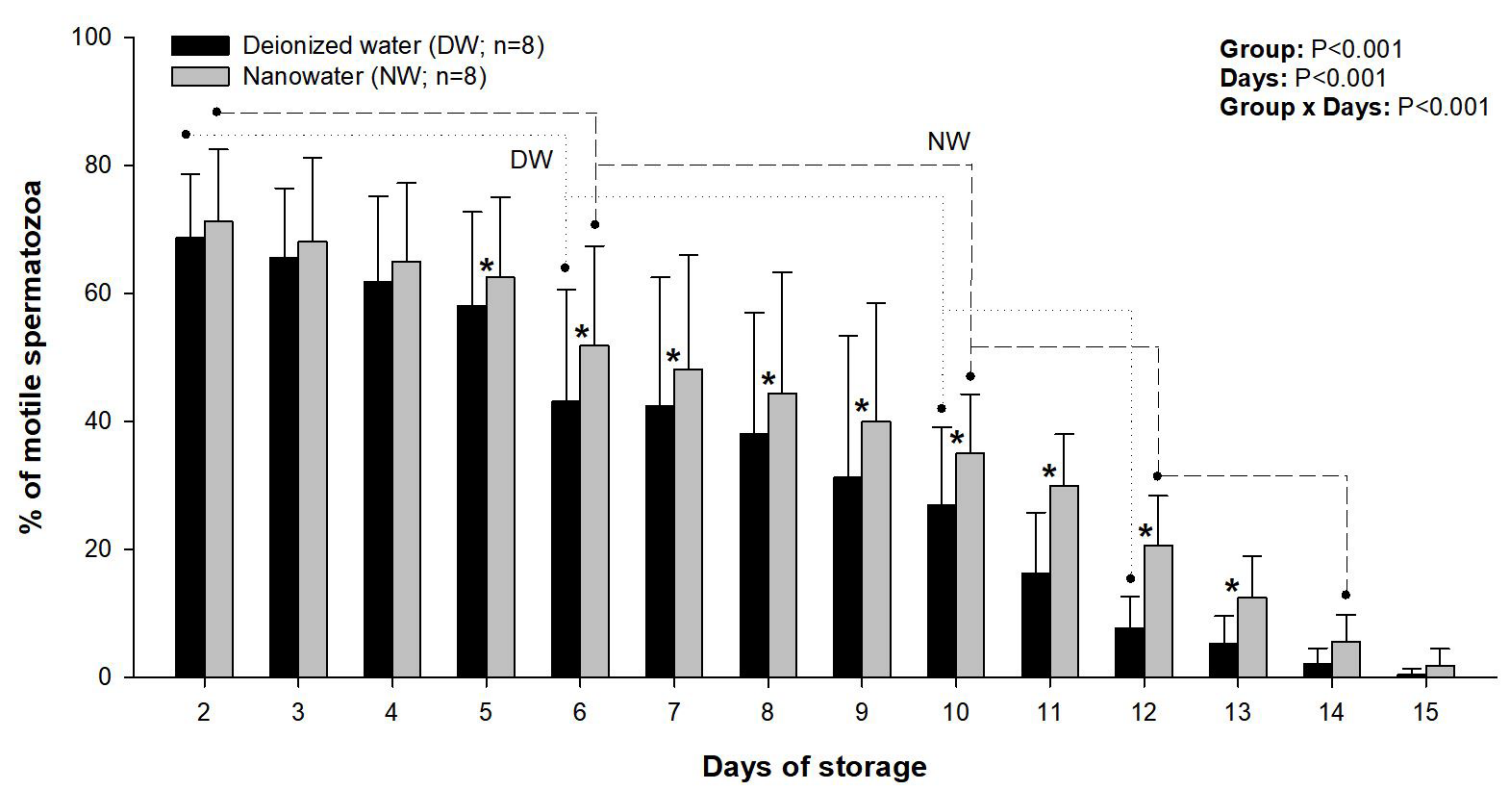
Changes in the percentage of progressively motile sperm (mean+SD) during the 15-day storage of boar semen in a commercial semen extender prepared with deionized water (DW) or nanowater (NW). Dotted and dashed lines denote successive significant decreases in mean values for DW and NW groups, respectively, and asterisks indicate significant differences between the two groups.

Sperm progressive motility decreased (P<0.05) between Days 2 and 6, Days 6 and 10, and Days 10 and 12 of storage in both groups (Figure 1). In addition, the percentage of motile sperm declined (P<0.05) from Day 12 to Day 14 in NW group. A decline in sperm progressive motility below 40% in all semen samples studied occurred on Day 11 in DW group and on Day 12 in NW group. From Days 5 to 13 of storage, the percentage of motile spermatozoa was greater (P<0.05) in NW group compared with DW group; during that period, a difference in the mean proportion of progressively motile sperm between the two groups ranged from 4 to 14%.

**Figure 2:**
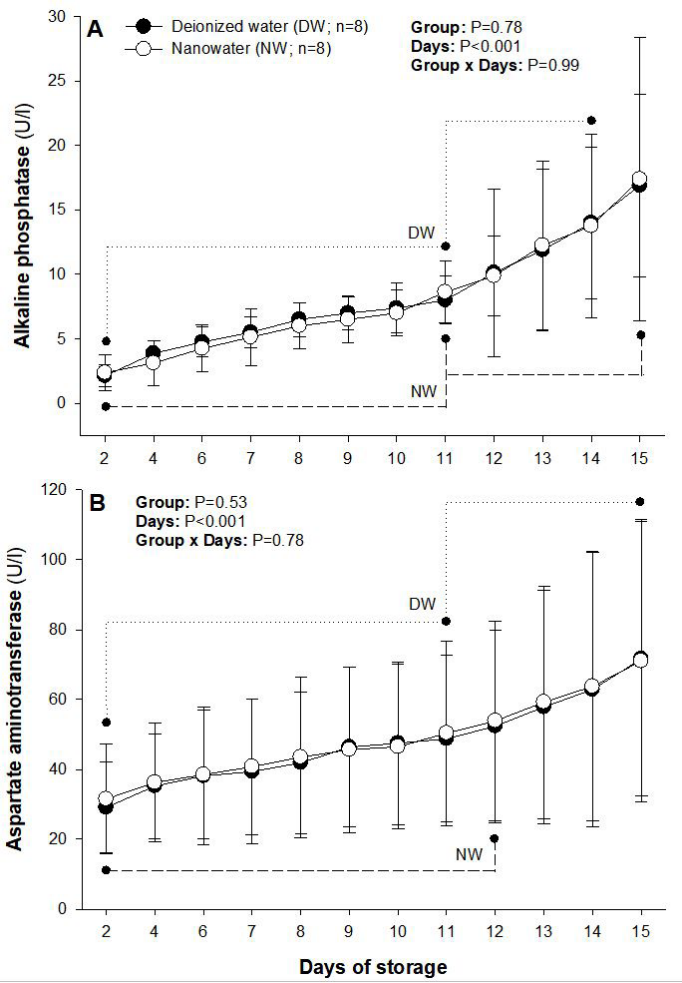
Mean (±SD) daily concentrations of alkaline phosphatase (ALP) (A) and aspartate aminotransferase (AspAT) (B) measured in extender samples during the 15 days of boar semen storage at 16-18 °C. Ejaculates were diluted to a final concentration of 3×10^9^ spermatozoa/ml in a commercially available, long-term semen extender prepared with deionized water (DW) or nanowater (NW). Dotted and dashed lines denote successive significant increases in enzyme content in DW and NW groups, respectively.

There were no significant differences in mean ALP and AspAT concentrations measured in extender samples during the 15 days of boar semen storage (Figure 2). ALP concentrations increased (P<0.05) from Day 2 to Day 11 in both groups and then between Days 11 and 14 or between Days 11 and 15 in DW or NW groups, respectively (Figure 2A). Mean AspAT concentrations increased (P<0.05) from Days 2 to 11 and then more rapidly (P<0.05) to Day 15 in DW group, whereas in NW group they rose between Days 2 and 12 (P<0.05; Figure 2B).

There were no significant relationships among semen dilution and morphological defects or sperm viability (i.e., survival times and decline in sperm progressive motility to ≤40%). The relative volume of ejaculate in extended semen was correlated negatively with sperm progressive motility on Day 11 (r=−0.88, P=0. 004) in NW group. The proportion of ejaculate in diluted boar semen was also correlated with ALP concentrations in DW group on Day 4 (r=−0.78, P=0.02) and in NW group on Days 11 (r=−0.77, P=0.03) and 12 (r=−0.71, P=0.05) as well as with AspAT concentrations in NW group on Day 15 of storage (r=−0.71, P=0.05).

## Discussion

There are two main factors that can diminish the viability of boar sperm preserved in a liquid state: the temperature at which semen is collected and then stored after dilution (i.e., a potential “cold shock”), and the amount and quality of semen extender used (i.e., the “dilution effect”). While the consequences of the cold shock on boar semen have been thoroughly described (Pursel et al., 1972; Johnson et al., 2000), the specific implications of the dilution factor remain poorly understood (Harrison et al., 1982). It has been suggested that the type and composition of semen extender may affect the functioning of several intracellular compartments leading to the changes in phospholipid membrane fluidity, ionic balance and metabolism, and consequently to sperm motility and lifespan. Our results show that the addition of nanowater (NW) to a commercial semen extender may partially ameliorate degenerative changes in boar spermatozoa during the long-term liquid storage.

The most prevalent morphological defect and the only one that was significantly reduced in NW compared with DW group was the occurrence of boar spermatozoa with mid-piece defects on the 5^th^ and 10^th^ day of storage. Originally, this type of defect develops when sperm cells stored in the tail of epididymis are exposed to abnormal epididymal secretions; these secretions are mainly controlled by testosterone and so any stressor (e.g., cold, inflammation, etc.) that affects testosterone production and bioavailability may result in mid-piece deformations (Watson, 2000). During liquid semen storage, the low availability of various seminal plasma constituents appears to be a primary cause of mid-piece defects (Aziz et al., 2011). Increased levels of reactive oxygen species (ROS) can be detected in semen samples with a high proportion of abnormal mid-pieces, and a positive correlation exists between mid-piece defects and various markers of sperm apoptosis (i.e., caspase-3) as well as progressing loss of the mitochondrial membrane potential/integrity (Aziz et al., 2011). Our results indicate that NW can exert cytoprotective effects on boar spermatozoa susceptible to degenerative changes induced by prolonged liquid storage. Whether or not the main mechanism of these protective actions of NW involves improved membrane transport and utilization of seminal plasma/extender constituents and/or neutralization of accumulating ROS remains to be elucidated.

There were no significant differences between the two types of extenders in mean concentrations of AspAT and alkaline ALP measured daily throughout the entire period of boar semen storage. AspAT is permanently bound to the sperm mid-piece membranes, and particularly to the mitochondrial membranes, and so its content typically reflects the structural and functional damage to the mid-piece area of the spermatozoon (Silva and Gadella, 2006). ALP is a dephosphorylating enzyme involved in glycolytic reactions that provide metabolic energy to generate mitochondrial ATP and sustain sperm function (Windsor, 1997). In this study, however, the increased incidence of mid-piece defects and significantly lower sperm progressive motility recorded between Days 5 and 13 of storage in DW group were not accompanied by a significant increase in the release of both enzymes, suggesting that the mid-piece defects could have “physical” (e.g., low temperature intolerance) rather than “biochemical” causes (e.g., low phospholipid content and instability of cell membranes). It is attractive to speculate that in addition to facilitated membrane transport of extender constituents (necessary for ATP production and maintenance of cellular integrity), NW could exert thermoprotective effects on boar spermatozoa stored in the liquid phase.

Our observations on sperm motility and survival times *ex situ* indicate that a rate of decline in sperm motility was more drastic than that in sperm survivability. Following the conversion of available energy sources to ATP, semen motility is dependent on the balance between Ca^+2^ and K^+^ ions governing hyperpolarization of the plasma membrane, and phosphorylation of proteins involved in the initiation and regulation of flagellar movements (Gagnon, 1990). Both processes appeared to be altered to a greater extent in DW group compared with NW group, leading to an earlier decline in sperm progressive motility to ≤40% at sperm survival tests.

Seminal plasma contains several substances that can contribute to sperm viability both in the female reproductive tract and during *ex vivo* processing and storage. Several seminal lipids (e.g., ethanolamine and choline ether lipids, and seminolipids essential for membrane raft function; Wood et al., 2016) and proteins (e.g., spermadhesins and non-heparin-binding PSP-I and PSP-II that account for >50% of all seminal plasma proteins; Gagnon, 1990) bind to the sperm surface. Semen extenders only contain the components needed for the metabolic maintenance of sperm cell activity (glucose), protection against cold shock (BSA), control of pH (bicarbonate, Tris, Hepes) and osmotic pressure (NaCl, KCl) of the storage medium, and inhibition of bacterial contamination (antibiotics; Gadea, 2003). Therefore, it was logical to assume that the proportion of ejaculate in extended semen would be directly related to the amount of seminal plasma-derived cytoprotective substances which, in turn, would have a positive effect on semen viability. In order to test this hypothesis, we examined semen characteristics determined in the present experiment for correlations with the proportion of ejaculate in inseminate doses diluted to a specific final concentration of spermatozoa. Contrary to our expectations, significant negative correlations were recorded on Days 4, 11-12 and 15 of storage and were confined to sperm progressive motility and ALP/AspAT concentrations. The influence of dilution factor on sperm characteristics was seen mainly in NW group. The inverse relationship between semen dilution and sperm progressive motility is puzzling but, in speculation, it can be explained by elevated activity of the protein inhibitors of sperm movement in less diluted semen samples (Gagnon, 1990). Further studies are necessary to better understand the relationship between the ejaculate dilution and the changes in sperm viability during the long-term liquid storage.

In summary, the motility and viability of boar spermatozoa stored in a commercial semen extender prepared with NW were greater than those in DW group. Furthermore, morphological defect rates, and particularly the percentage of mid-piece defects, were lower in the extender containing NW compared with those in the DW-diluted semen extender. These differences in sperm characteristics were not associated with significant variations in ALP and AspAT concentrations in storage medium suggesting that beneficial effects of NW could be, at least partly, due to its thermoprotective properties as well as the enhancement of transmembrane transport of various extender constituents. The proportion of ejaculate in extended semen was inversely related to semen motility and ALP/AspAT concentrations mainly in NW group. The specific mechanisms of the cytoprotective effects of NW and fertilizing ability of boar semen during the long-term liquid storage in the NW-containing extenders remain to be elucidated. Previous results of a field trial have indicated that the use of boar semen stored for 5 days in the NW-containing extender was associated with a consistent increase in the litter size and numbers of live-born piglets, and a concurrent decline in the number of stillborn piglets (Schwarz et al., 2014); the reproductive outcomes were compared to those obtained with boar semen extended according to manufacturer’s specifications. Those earlier observations and our present results warrant further studies of the fertilizing ability of boar semen preserved with the use of NW for >5 days.
